# A Pharmacological Investigation of Eph-Ephrin Antagonism in Prostate Cancer: UniPR1331 Efficacy Evidence

**DOI:** 10.3390/ph16101452

**Published:** 2023-10-13

**Authors:** Claudio Festuccia, Miriam Corrado, Alessandra Rossetti, Riccardo Castelli, Alessio Lodola, Giovanni Luca Gravina, Massimiliano Tognolini, Carmine Giorgio

**Affiliations:** 1 Department of Biotechnological and Applied Clinical Sciences, University of L’Aquila, Via Vetoio (Coppito), 67100 L’Aquila, Italy; alessandra.rossetti@univaq.it (A.R.); giovanniluca.gravina@univaq.it (G.L.G.); 2 Department of Food and Drug, University of Parma, Parco Area delle Scienze 27/A, 43124 Parma, Italy; miriam.corrado@unipr.it (M.C.); riccardo.castelli@unipr.it (R.C.); alessio.lodola@unipr.it (A.L.); massimiliano.tognolini@unipr.it (M.T.)

**Keywords:** Eph receptors, ephrin ligands, EphA2 antagonism, prostate cancer, PC3 xenograft mice

## Abstract

The Eph kinases are the largest receptor tyrosine kinases (RTKs) family in humans. PC3 human prostate adenocarcinoma cells are a well-established model for studying Eph–ephrin pharmacology as they naturally express a high level of EphA2, a promising target for new cancer therapies. A pharmacological approach with agonists did not show significant efficacy on tumor growth in prostate orthotopic murine models, but reduced distal metastasis formation. In order to improve the comprehension of the pharmacological targeting of Eph receptors in prostate cancer, in the present work, we investigated the efficacy of Eph antagonism both in vitro and in vivo, using UniPR1331, a small orally bioavailable Eph–ephrin interaction inhibitor. UniPR1331 was able to inhibit PC3 cells’ growth in vitro in a dose-dependent manner, affecting the cell cycle and inducing apoptosis. Moreover, UniPR1331 promoted the PC3 epithelial phenotype, downregulating epithelial mesenchymal transition (EMT) markers. As a consequence, UniPR1331 reduced in vitro PC3 migration, invasion, and vasculomimicry capabilities. The antitumor activity of UniPR1331 was confirmed in vivo when administered alone or in combination with cytotoxic drugs in PC3-xenograft mice. Our results demonstrated that Eph antagonism is a promising strategy for inhibiting prostate cancer growth, especially in combination with cytotoxic drugs.

## 1. Introduction

The Eph kinases are the largest receptor tyrosine kinases (RTKs) family in humans. The sixteen members identified so far belong to two different classes (A and B), based on the sequence homology and binding affinity for their ephrin ligands. As Eph receptors, ephrins are also classified into A and B subclasses, where the former has a glycosyl-phosphatidylinositol linkage to the membrane, whereas the latter contains a transmembrane domain. Generally, upon Eph–ephrin binding, bidirectional signals are triggered: a forward signal from the Eph receptors and a reverse signal from the ephrin ligands are generated into the cells [1]. Besides the so-called canonical activation, requiring a physical interaction between opposite cells, a multitude of other patterns of activation have been reported in the literature [2].

The Eph–ephrin system is physiologically involved in the proliferation and differentiation of progenitor cells in the embryo, participating in the regulation of morphogenesis. The same activity has been demonstrated in adult stem cells, where it contributes to the maintenance of the regenerative capacities and homeostasis of some tissues, including the intestines [3], nervous system [4], breast [5], and skin [6]. Minor roles in insulin secretion [7] and platelet aggregation have been also reported [8].

In the last two decades, a prominent role of this system in cancer has also been disclosed by an extensive study describing its involvement in the immunosuppressive tumor microenvironment [9], tumor angiogenesis, cell motility, tumor growth, and metastasis [10,11,12]. Depending on cellular context and tumor type, Eph receptors and ephrin ligands can act as both tumor promoters and tumor suppressors in several solid tumors, including prostate cancer (see below). For these reasons, some academic groups and companies have been actively looking for a targeting strategy based on biologics and small molecules, with a focus on agents working as kinase inhibitors, receptor agonists and antagonists, and protein–protein interaction (PPi) inhibitors [13]. Many pharmacological tools targeting the Eph–ephrin system have been discovered and proven their activity in many preclinical models. Recently, Tröster and colleagues summarized the rational use of EphA2 kinase inhibitors, such as NVP-BHG712, GLPG1790, and ALW-II-41-27, in cetuximab-resistant colon rectal cancer [14]. Instead, anti-EphA5 mAb 11C12 and Eph and ephrin recombinant proteins, together with tyrosine kinase inhibitors targeting Eph receptors such as Dasatinib and Osimertinib, have shown promising results in preclinical models of lung cancer [15]. These reviews are the most recent works highlighting the potential use of Eph/ephrin targeting in cancer therapy. Indeed, in the literature, we can find different pharmacological approaches to tackling solid tumors beyond colon and lung cancer [16].

Since 2011, we have been devoting our work to this topic: we identified lithocholic acid as an hit compound [17], and then a series of derivatives was discovered peaking in the synthesis of UniPR139 and UniPR502, a pair of potent and bioavailable pan-Eph–ephrin interaction inhibitors [18]. Moreover, starting from lithocholic acid, a drug discovery program combining target hopping with a ligand- and structure-based approach led to the discovery of UniPR1331 [19,20].

PC3 human prostate adenocarcinoma cells are a well-established model for studying Eph–ephrin pharmacology as they naturally express a high level of EphA2, the most promising target in cancer within Eph receptors and ephrin ligands [21]. Targeting the Eph–ephrin system in prostate cancer was previously focused on EphA2 activation, followed by its internalization and degradation through the use of agonists. This strategy was described for the first time by Petty and colleagues using the agonist small molecule doxazosin [22], and, more recently, by Pellecchia’s group using an agonist dimeric synthetic agent [23]. Both the research groups concluded that the pharmacological approach with agonists did not show significant efficacy on tumor growth in prostate orthotopic murine models, but was able to reduce the formation of distal metastasis. To improve the comprehension of the pharmacological targeting of Eph receptors in prostate cancer, we designed and performed a set of experiments aimed at assessing the efficacy of Eph antagonism in a xenograft murine model of prostate cancer based on PC3 cells. To this end, we used UniPR1331 (alone and in combination with cytotoxic drugs), a small PPi inhibitor of the Eph–ephrin system, that has recently been proven to significantly reduce the tumor growth in different models of glioblastoma [24].

## 2. Results

### 2.1. UniPR1331 Inhibited PC3 Cells’ Growth In Vitro

**Figure 1:**
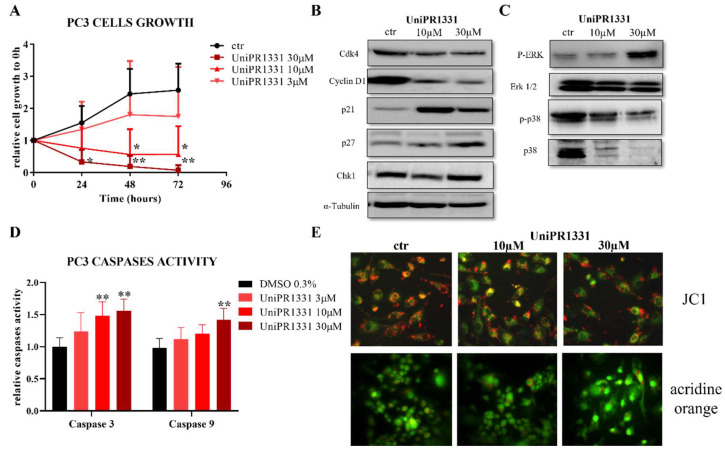
(**A**) Cell growth of PC3 cells treated with UniPR1331 at 30, 10, and 3 µM for 24, 48, and 72 h. Two-way ANOVA followed by Tukey’s post-test was used to compare all the groups to control group (DMSO 0.3%) at each time point, * *p* < 0.05 ** *p* < 0.01. (**B**) Western blot analysis of UniPR1331-treated PC3 cells for cyclin D1, CDK4, p21, p27, and chK1, as well as for ERK1/2/p-ERK and p38/p-p38 (**C**). (**D**) Enzymatic detection of caspases activity 3 and 9 in UniPR1331-treated PC3 cells. For both the caspases, one-way ANOVA followed by Dunnett’s post-test was used to compare all the groups to control group (DMSO 0.3%), ** *p* < 0.01. (**E**) JC1 (apoptosis index) and acridine orange (autophagy index) staining of UniPR1331-treated PC3 cells.

Before testing UniPR1331 in PC3-xenograft mice, the potential antitumor activity of this compound was studied in vitro by the means of an MTT vitality assay. A proliferation assay was performed on PC3 cells treated with UniPR1331 or DMSO 0.3% for 24, 48, and 72 h. As shown in Figure 1A, UniPR1331 inhibited the PC3 cells’ growth in a dose-dependent manner. In order to investigate in which way UniPR1331 could inhibit the PC3 cells’ growth, we performed a series of Western blots on cell lysates from PC3 cells incubated with UniPR1331 at the antiproliferative concentrations of 30 µM and 10 µM. As shown in Figure 1B, UniPR1331 affected the PC3 cell cycle by inhibiting the expression of inducible protein cyclin D1 and, to a lesser extent, the expression of the constitutive protein CDK4. According to these data, p27 and especially p21 resulted in being overexpressed in the UniPR1331-treated PC3 cells. Conversely, the chk1 protein appeared to be unaffected by UniPR1331. Moreover, Western blot analyses showed that UniPR1331 affected the MAPK pathways when incubated with the PC3 cells, promoting ERK1/2 phosphorylation and p38 inhibition (Figure 1C). The increased activity of caspase 3 and 9 in the UniPR1331-treated PC3 cells suggested that UniPR1331 inhibits PC3 cells’ growth by promoting apoptosis (Figure 1D). JC1 staining (apoptosis index) confirmed molecular biological data about the promotion of apoptosis by UniPR1331, whilst acridine orange staining (autophagy index) revealed that UniPR1331 does not induce PC3 cells’ autophagy.

### 2.2. UniPR1331 Did Not Cause the EphA2 Internalization in PC3 Cells

**Figure 2:**
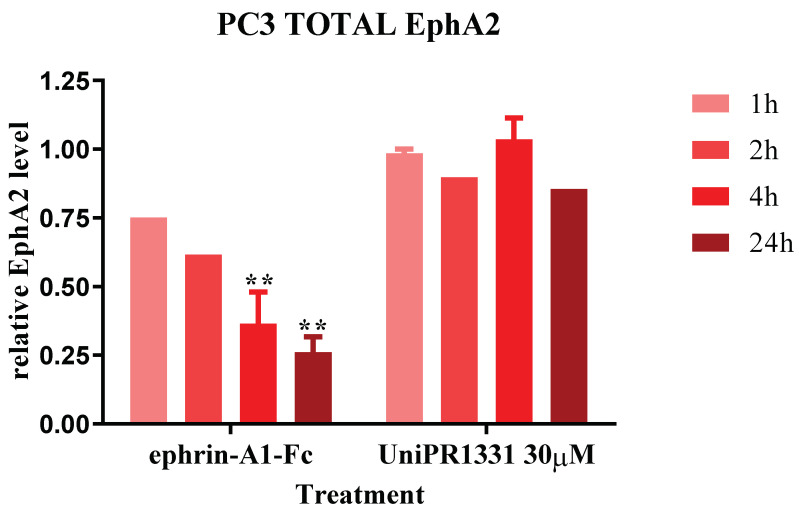
The EphA2 levels of PC3 cells stimulated with ephrin-A1-Fc 0.25 µg/mL or treated with UniPR1331 30 µM are relative to untreated PC3 cells. For both the treatments, one-way ANOVA followed by Dunnett’s post-test were used to compare all the groups to untreated group, ** *p* < 0.01.

Ephrin-A1-Fc, the physiological ligand of EphA2, is able to cause the internalization and degradation of the receptor upon its activation [25]. Previous works, targeting the EphA2 receptor with specific antibodies and agonist compounds, have associated the antitumor effect of these molecules with their ability to reduce EphA2 membrane levels upon their binding and activation of the receptor. Therefore, it was investigated if UniPR1331, as a binder of the EphA2 receptor, could evoke the same response. UniPR1331 did not reduce the relative level of EphA2 at 30 µM over time, whereas ephrin-A1-Fc 0.25 µg/mL, used as a positive control, caused a decrease in the receptor levels (Figure 2). These data suggest that the internalization and degradation of the EphA2 receptor depend on its activation rather than its simple interaction with an antagonist such as UniPR1331. Our results also showed that UniPR1331 is able to inhibit PC3 cells’ growth, inhibiting EphA2 activity without causing its degradation.

### 2.3. UniPR1331 Inhibited PC3 Epithelial–Mesenchymal Transition (EMT) and Promoted Epithelial Phenotype

**Figure 3:**
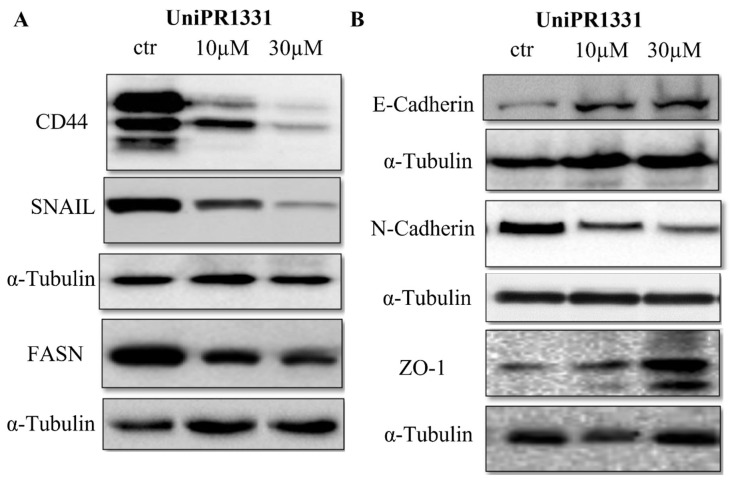
(**A**) Western blotting analysis of UniPR1331-treated PC3 cells for CD44, SNAIL and FASN. (**B**) Western blotting analysis of UniPR1331-treated PC3 cells for E-Cadherin, N-Cadherin and ZO-1.

The stemness maintenance and acquisition of an invasive phenotype by the means of the epithelial–mesenchymal transition are key steps in cancer progression, including prostate cancer [26]. As is shown in Figure 3, a Western blot analysis of lysates from the UniPR1331-treated PC3 cells revealed a reduction in the PC3 stem marker CD44 and the inhibition of EMT markers’ expression. UniPR1331 promoted E-cadherin expression and reduced N-cadherin levels, highlighting its ability to inhibit the PC3 mesenchymal phenotype. According to these data, the suppressor transcriptor factor zinc finger protein SNAI1 (SNAIL) resulted in being inhibited by UniPR1331. Indeed, SNAIL promotes the repression of E-cadherin and is considered to be an EMT marker [27]. Moreover, UniPR1331 was able to improve the expression of tight junction protein-1 (ZO-1), confirming the ability of UniPR1331 to maintain PC3 cells in a differentiate state. In this view, it is noteworthy how the UniPR1331 treatment was able to reduce the expression of the fatty acid synthase (FASN), as the dysregulation of lipid metabolism via FASN overexpression is considered to be a hallmark of prostate cancer progression [28].

### 2.4. UniPR1331 Inhibited PC3 Migration, Invasion, and Vasculomimicry Capabilities

**Figure 4:**
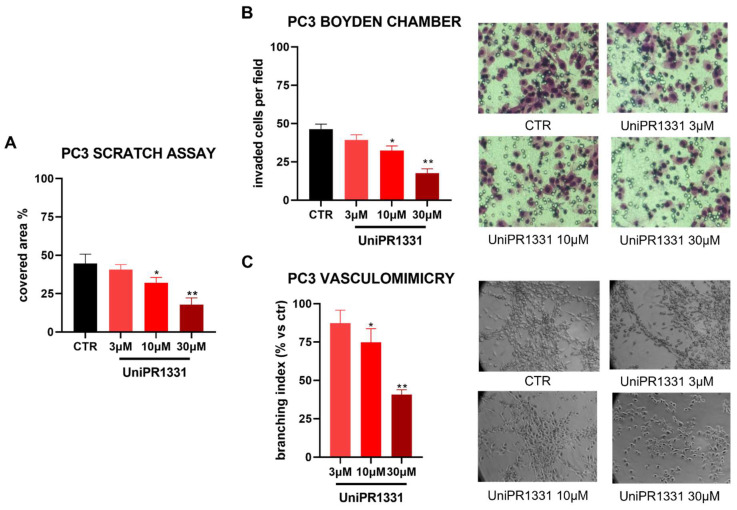
(**A**) Migration, (**B**) invasion, and (**C**) vasculomimicry capabilities of PC3 cells treated with UniPR1331 at 30, 10, and 3 µM. One-way ANOVA followed by Dunnett’s post-test was used to compare all the groups to control group (DMSO 0.3%), * *p* < 0.05 ** *p* < 0.01.

It is well established that EMT increases the migration and invasion capabilities of cancer cells, including in prostate cancer [29]. The detrimental effects of UniPR1331 on PC3 EMT markers prompted us to investigate if UniPR1331 treatment could affect PC3 migration, invasion, and vasculomimicry capabilities. As shown in Figure 4A, UniPR1331 was able to inhibit, in a concentration-dependent manner, PC3 migration, as well as the Matrigel invasion (Figure 4B). Finally, the incubation of UniPR1331 with PC3 seeded on Matrigel inhibited the vasculomimicry process in a concentration-dependent manner (Figure 4C).

### 2.5. UniPR1331 and the Associations with Cytotoxic Drugs Reduced the Cancer Growth in PC3-Xenograft Mice

**Figure 5:**
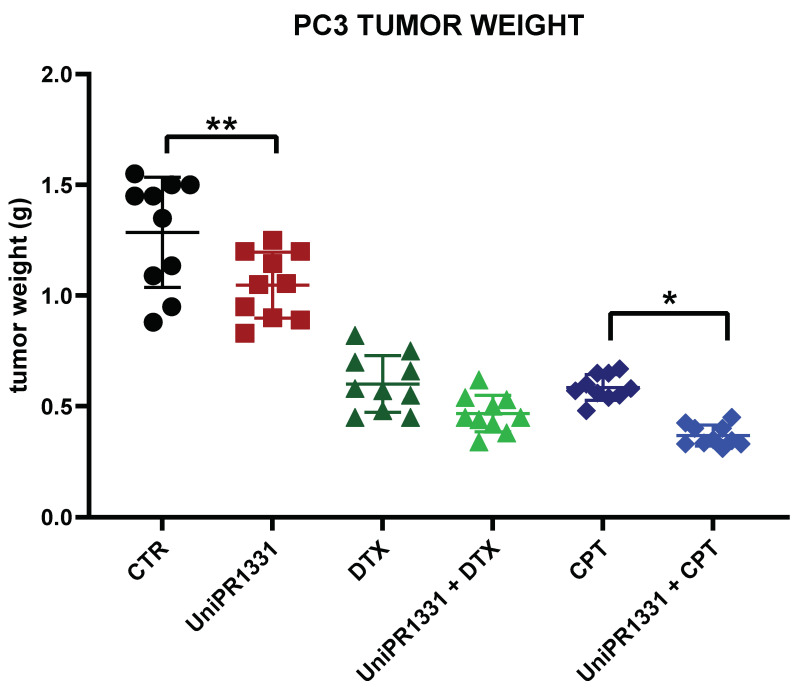
Comparisons amongst treatments in terms of final tumor weight in PC3-xenograft nude mice after 5 weeks of treatment. One-way ANOVA followed by Tukey’s post-test was used to compare all the groups to control group, * *p* < 0.05, ** *p* < 0.01. CTR was significantly different from any treatment or association and UniPR1331 was significantly different from DTX, CPT, and their associations (data not reported in the graph for simplicity). All the groups passed Shapiro–Wilk normality test.

As UniPR1331 was able to inhibit the PC3 cells’ growth and reduce the PC3 migration, invasion, and vasculomimicry capabilities in vitro, we tested its efficacy in a model of prostate cancer induced by PC3 xenografted in nude mice. The daily administration of 30 mg/kg/os of the compound in the PC3-xenograft mice reduced the tumor weight by 23% when compared to the control (Figure 5). The tumors reached a final weight of 1.05 g (95% c.l. 0.94–1.15 g) in the UniPR1331 group and 1.29 g (c.l. 1.11–1.47 g) in the control group after 35 days of treatment. When tested alone, UniPR1331 proved to be less effective than Docetaxel (DTX) and Cisplatin (CPT) monotherapy, which showed a marked reduction in the tumor weight: 0.60 g for DTX (c.l. 0.51–0.69 g) and 0.59 g for CPT (c.l. 0.54–0.63 g). Nevertheless, the association between UniPR1331 and CPT significantly reduced the final tumor weight by an additional 34% when compared to CPT alone, resulting in a final weight of 0.37 g (c.l. 0.33–0.40 g) vs. 0.59 g (c.l. 0.54–0.63 g). Conversely, the association with DTX was well tolerated, but showed no significant therapeutic benefit when compared to DTX alone. The UniPR1331 ability to reduce the PC3 stem marker CD44 gave us the rationale to investigate the association with CPT and DTX, due to the critical role played by prostate cancer stem cells in chemoresistance [30].

## 3. Discussion

Since 2006, when Fox and colleagues compared the transcripts of all Eph receptors and ephrin ligands in several prostate cell lines, ranging from cultures obtained by the normal prostate epithelium to cell lines established by prostate tumors of varying degrees of metastasis, the possible key role of Eph–ephrin signaling in the development of prostate cancer was highlighted [31]. For this reason, it is not surprising that newly synthesized molecules, endowed with agonist [23] or antagonist [32] properties, were pharmacologically characterized in prostate cell lines and, in particular, in PC3 cells, naturally overexpressing EphA2, the most studied Eph receptor in cancer. In the last decade, several and different pharmacological approaches have been proposed to target this system in prostate cancer. In the earliest studies, it was shown how EphA2 activation without degradation by the means of low concentrations of ephrin-A1-Fc was a key way of efficiently inhibiting prostate tumor growth, at least in vitro [33]. Then, this view was broadened when Tawadros and colleagues showed that the EphA2 receptor was able to sustain prostate cancer cells’ migration through endothelial barrier in a ligand-independent way, suggesting that the degradation of the EphA2 receptor could be a valid strategy in metastasis inhibition [34]. According to these observations, different in vivo experimentations with agonist molecules of the EphA2 receptor, causing the activation and degradation of the receptor, have shown the inhibition of prostate metastasis formation [22,23].

In the present work, UniPR1331, an Eph–ephrin antagonist, was shown to inhibit, in a dose-dependent manner, PC3 cells’ growth in vitro, arresting the PC3 cell cycle in the G_0_/G_1_ phase and blocking the cyclin D1-CDK4 complex. Moreover, Western blot analyses showed that UniPR1331 affected the MAPK pathways when incubated with PC3 cells. Although ERK1/2 is a pro-survival factor contributing to cell proliferation and differentiation, it was demonstrated that, under some circumstances, it can also promote apoptosis in several types of cells, including PC3. Indeed, several works have demonstrated that esogenous molecules can induce PC3 cells’ apoptosis by the means of ERK1/2 activation and p38 inhibition [35]. The increased activity of caspase 3 and 9 in the UniPR1331-treated PC3 cells suggested that also UniPR1331 inhibited the PC3 cells’ growth, promoting apoptosis by the means of ERK1/2 phosphorylation and p38 inhibition. JC1 staining confirmed molecular biological data, as UniPR1331 caused mitochondrial depolarization (apoptosis index) in a concentration-dependent manner. Indeed, when UniPR1331 was incubated with the PC3 cells, the ratio between JC1 red aggregates and JC1 green monomers decreased, highlighting a reduction in mitochondrial membrane potential. Conversely, acridine orange staining (autophagy index) revealed that UniPR1331 did not promote the formation of acidic vesicles organelles when incubated with the PC3 cells, suggesting that, as UniPR1331 does not induce autophagy, in treated cancer cells, it can represent a cellular defense mechanism [36] and its inhibition an antitumor effect besides apoptosis [37].

Unlike agonist molecules, UniPR1331 did not modify the membrane levels of EphA2 up to 30 µM after 24 h of incubation, suggesting that the activation of the EphA2 receptor is essential for its degradation and that the PC3 cells’ growth could be reduced by inhibiting the Eph–ephrin interaction without affecting the membrane levels of EphA2, at least in vitro. Therefore, EphA2 binders can inhibit cancer cells’ growth blocking EphA2 activity both by the means of its degradation (agonist binders) and by the means of the inhibition of Eph–ephrin interactions (antagonist binders).

Moreover, a series of Western blot analyses showed that UniPR1331 was able to maintain the PC3 cells in a differentiate epithelial state, reducing the expression of the PC3 EMT markers N-cadherin, SNAIL, and FASN. Conversely, UniPR1331 increased the expressions of E-cadherin and ZO-1. These data were corroborated by functional assays, in which UniPR1331 was able to inhibit the migration invasion and vasculomimicry capabilities of the PC3 cells.

The antitumor activity of UniPR1331 was then confirmed in vivo, when the PC3 cells were engrafted in the nude mice. Indeed, the daily administration of 30 mg/kg/os of the compound in the PC3-xenograft mice slightly but significantly reduced the tumor weight by 23% after 35 days of treatment when compared to the controls. We obtained the most promising results when we investigated the combination of UniPR1331 with cisplatin, due to the ability of UniPR1331 to reduce the PC3 stem marker CD44 and the well-known role of prostate cancer stem cells in chemoresistance [31]. Indeed, the combination with cytotoxic drugs showed that UniPR1331 was able to improve the activity of cisplatin in a significant manner. Cytotoxic drugs are used in hormone-resistant prostate cancer and PC3 cells are considered in castration-resistant prostate cancer cell lines [38]. Therefore, our results suggest that Eph antagonism in association with cytotoxic drugs could be a possible pharmacological approach in hormone-resistant prostate cancer. In future, it would be interesting to evaluate in the same model the association of UniPR1331 with different doses of cytotoxic drugs, focusing not only on the efficacy of the combination, but also on its tolerability. In conclusion, our work demonstrated that UniPR1331 is able to partially inhibit prostate tumor growth in a PC3-xenograft model as a single agent and is a promising adjuvant in chemotherapy. Finally, Miao and colleagues demonstrated that EphA2–ephrin–A1 interaction strengthened adhesion to collagen I, explaining how the Eph–ephrin system may not only have an important role in the detachment of cancer cells from the primary tumor, but also in the process of bone metastasis formation [39]. PC3 cells were isolated from a patient with bone metastatic prostate cancer in 1979 [40]. Amongst the prostate cancer cell lines, PC3 cells show a high metastatic potential and are commonly used in in vivo experimentations due to their highly aggressive nature [41]. According to our preliminary in vitro data on PC3 migration and invasion assays, it would be interesting to evaluate the efficacy of UniPR1331 in a mouse model of PC3 bone metastasis, which occur in more than 80% of advanced-stage prostate cancer [42].

## 4. Materials and Methods

### 4.1. PC3 Culture

The PC3 cells (ECACC, Portdown, UK) were cultured in HAM’S NUTRIENT MIXTURE F-12 with L-Glutamine, fetal bovine serum (FBS) 7%, and penicillin/streptomycin 1%. Medium, FBS, and antibiotic solution were purchased from EuroClone. The cells were grown in a humidified atmosphere of 95% air and 5% CO_2_ at 37 °C. The cell line was grown in a humidified atmosphere of 95% air and 5% CO_2_ at 37 °C. The cell line was authenticated on 17 November 2020 and on 29 September 2021 (Eurofins Genomics, Louisville, KY, USA) [43].

### 4.2. MTT Assay

In total, 7 × 10^3^ PC3 cells per well were seeded in 96-well plates. The day after, the growth medium was replaced with fresh medium and treated with UniPR1331 for 24, 48, and 72 h. Then, the wells were emptied and MTT 1 mg/mL of solution was added and incubated for 2 h. The resulting formazan crystals were solubilized with DMSO and the developed color was measured using an ELISA plate reader. The results of three separate experiments are presented as the mean ± SD.

### 4.3. Human Total EphA2 ELISA

A total of 10^5^ PC3 cells per well were seeded in 6-well plates. The day after, the growth medium was replaced with fresh medium without FBS and treated with ephrin-A1-Fc 0.25 µg/mL, UniPR1331 30 µM, and PBS or DMSO 0.3% for 1, 2, 4, and 24 h. Then, the cell lysates were collected and the EphA2 levels were detected by the means of Human Total EphA2 ELISA DUOSET^®^ IC (R&D system, Minneapolis, MN, USA).

### 4.4. Preparation of Cell Lysates and Western Blot Analysis

The PC3 cells were seeded and grown in 90 mm diameter Petri dishes. After treatment, the cells were washed with cold PBS and immediately lysed with 1 mL of lysis buffer containing a protease and phosphatase inhibitors cocktail. Cytosol and lysosome fractions were obtained by using the nuclear/cytosol fractionation and lysosome purification kit (Biovision, Inc., Milpitas, CA, USA). The total lysates and sub-fractionated extracts were (i) electrophoresed in 10% SDS-PAGE. The separated proteins were transferred to nitrocellulose membrane and probed with the appropriate antibodies using the conditions recommended by the suppliers. The total extracts were normalized by using an anti α-tubulin antibody. (ii) The total lysates and sub-fractionated extracts were also exploited in ELISA assays.

### 4.5. JC1 Staining and Acridin Orange Staining

JC1 staining: the membrane-permeant green fluorophore JC-1 (Thermofisher, Waltham, MA, USA) was used in apoptosis studies to evaluate mitochondrial health. JC-1 dye exhibits potential-dependent accumulation in mitochondria as red fluorescent JC1-aggregates. Mitochondrial depolarization is indicated by a decrease in the red/green fluorescence intensity ratio.

Acridin orange: the acridine orange solution (Sigma Aldrich, Burlington, MA, USA) was used in autophagy studies to evaluate acidic vesicles organelles formation. Acridine Orange solution is a cell-permeable green fluorophore that can be protonated and trapped in acidic vesicular organelles, where its accumulation promotes a red fluorescence emission. The presence of an orange/red staining is indicative of acidic vesicular organelles formation.

### 4.6. Scratch Assay

In total, 5 × 10^5^ PC3 cells per well were seeded in 6-well plates. After 24 h, we did a scratch in each well and then we treated the PC3 cells with UNIPR1331 or DMSO 0.3% as a control. With the use of the IncuCyte^®^ S3 (Sartorius, Göttingen, Germany), we collected a series of images every 3 h until 18 h. The images were analyzed through the use of the software ImageJ.

### 4.7. Boyden Chamber

An invasion test was performed as previously described using a 48-well Boyden chamber containing 8 μm polycarbonate filters (Neuroprobe, Inc., Gaithersburg, MD, USA). An invasion assay was performed in invasion chambers containing a membrane coated with Matrigel™. Untreated or UniPR1331-treated PC3 cells were added (75,000 cells/50 μL) to the top of each chamber and allowed to invade through coated filters for 6 h. In the lower compartment of the invasion chamber, 7% FBS-containing medium was added as a chemo-attractant. At the end of the incubation, the migrated/invaded cells attached to the lower membrane surface and were fixed, stained with Diff Quick (MBT), and counted with standard optical microscopy. The results of three separate experiments are presented as the mean ± SD.

### 4.8. Vasculomimicry (VM)

The PC3 cells were maintained for 24 h in serum-free medium and then seeded 1.5 × 10^4^ cells/well in µslides 15 wells (Ibidi, Gräfelfing, Germany) covered with Matrigel™. After treatment (UniPR1331 at different concentrations vs. DMSO 0.3%), the degree of VM response was evaluated using an inverted phase-contrast microscope. Each well was photographed and the relevant acquired images were analyzed using the ImageJ analysis software to calculate the number of vessels and the branching index. The results of three separate experiments are presented as the mean ± SD.

### 4.9. Xenograft Model

Six-week-old male CD1-nu/nu mice (Charles River, Milan, Italy) were maintained under the guidelines established by our Institutions (University of L’Aquila, Medical School and Science and Technology) and complying with the Italian government regulation n. 26, 4 March 2014 for the use of laboratory animals. All the mice received a subcutaneous injection of 1 × 10^6^ PC3 cells for each flank. The animal experiments received the approval of the local Animal Care Committee and Italian Ministry of Health (approval code 555/2017-PR) and the mice were used in compliance with the European Community Council Directive 2010/63/UE and Italian regulation (DL 26/2014).

### 4.10. Treatments for In Vivo Experiments

The animals were randomized into different groups and treatment started when the tumors reached a mean volume of 200 mm^3^, calculated using a Vernier caliper and the following formula: tumor volume (mm^3^) = d^2^ × D/2, where d and D are the shortest and longest diameters, respectively. The animals were treated with saline, 30 mg/kg/d per os UniPR1331, 7.5 mg/kg/w ip docetaxel (DTX), 5 mg/kg/d per os cisplatin (CPT), and associations of UniPR1331 with CPT or DTX.

## Data Availability

Data is contained within the article.
